# Gastrointestinal parasite infections in Nepalese Gurkha recruits arriving in the United Kingdom from 2012–2020

**DOI:** 10.1371/journal.pntd.0011931

**Published:** 2024-01-26

**Authors:** William D. Nevin, Jayne Jones, Donna Tupper, James A. T. Dunbar, Duncan Wilson, David Ross, Stephen Woolley, James Dodd, Jason Biswas, Lucy Lamb, Nicholas J. Beeching, Matthew K. O’Shea, Thomas E. Fletcher

**Affiliations:** 1 Department of Clinical Sciences, Liverpool School of Tropical Medicine, Pembroke Place, Liverpool, United Kingdom; 2 Department of Infectious Diseases, Imperial College London, United Kingdom; 3 Clinical Diagnostic Parasitology Laboratory, Liverpool School of Tropical Medicine, United Kingdom; 4 Medical Centre, Infantry Training Centre, Catterick, United Kingdom; 5 Friarage Hospital, Northallerton, United Kingdom; 6 212 Field Hospital, Royal Army Medical Corps, Defence Medical Services, United Kingdom; 7 Headquarters Defence Medical Services Group, Defence Medical Directorate, ICT Building, Edgbaston, Birmingham, United Kingdom; 8 Defence Public Health Unit, Defence Medical Services, United Kingdom; 9 Southmead Hospital, North Bristol NHS Trust, Bristol, United Kingdom; 10 Academic Department of Military Medicine, Royal Centre for Defence Medicine, Queen Elizabeth Hospital Birmingham, United Kingdom; 11 Department of Infectious Diseases, Royal Free Hospital, London, United Kingdom; 12 Centre of Defence Pathology, Royal Centre for Defence Medicine, Queen Elizabeth Hospital Birmingham, Edgbaston, Birmingham, United Kingdom; 13 Institute of Immunology and Immunotherapy, College of Medical & Dental Sciences, University of Birmingham, Edgbaston, Birmingham, United Kingdom; James Cook University, AUSTRALIA

## Abstract

**Background:**

Gastrointestinal parasite (GIP) infections are a major cause of global morbidity, infecting hundreds of millions of people each year and potentially leading to lifelong infection and serious complications. Few data exist on screening for GIP infections in migrants entering the UK or on the current performance of different traditional diagnostic approaches.

This study aimed to describe the prevalence of GIP infections in Nepalese Gurkha recruits screened on arrival in the UK.

**Methodology/Principal findings:**

We present a retrospective analysis of data from screening male adults (18–21 years) who arrived in the UK from Nepal between 2012 and 2020. Three separate faecal samples were obtained from participants at weekly intervals and processed for formalin-ethyl acetate (FEA) concentration/light microscopy and charcoal culture. Serum samples were analysed for IgG antibodies to *Strongyloides stercoralis* by ELISA.

Results were available from 2,263 participants, of whom 463 (20.5%, 95% CI 18.8%-22.2%) had a positive diagnostic test for at least one GIP infection. A total of 525 potential infections were identified. *Giardia duodenalis* was most common (231/2263, 10.2%), followed by *S*. *stercoralis* (102/2263, 4.5%), and hookworm species (86/2263, 3.8%). Analysis (microscopy and culture) of the initial stool sample diagnosed only 244/427 (57.1%) faecally identified pathogens, including 41/86 (47.7%) hookworm infections. The proportion of participants infected with any GIP showed a downward trend over the study period. Log-binomial regression showed risk of infection decreasing by 6.1% year-on-year (95% CI 3.2% - 9.0%). This was driven predominantly by a fall in hookworm, *S*. *stercoralis* and *Trichuris trichiura* prevalence.

**Conclusions/Significance:**

The level of potentially pathogenic GIP infection in young Nepalese men migrating to the UK is high (20.5%) and requires a combined diagnostic approach including serology and analysis of multiple stool samples incorporating specialised parasitological methods. Advances in molecular approaches may optimise and simplify the intensive screening strategy required.

## Author summary

Gastrointestinal parasite (GIP) infections caused by protozoa and helminths affect hundreds of millions of people worldwide, resulting in significant morbidity, but remain neglected in terms of diagnosis and management. Migrant and refugee populations are rarely offered screening for GIP infection in the UK, but providing this can create opportunities for treatment, as well as to gather valuable epidemiological data. We present a retrospective analysis of screening for gastrointestinal parasites in 2,263 young Nepalese men arriving in the UK as military Gurkha recruits, using a range of conventional diagnostic methods. The presented study is one of the largest reported and most complete approach to GIP screening in a homogeneous migrant population, finding high rates of GIP infection. When conventional diagnostic methods of light microscopy and charcoal culture are used, we found serial stool collection in combination with serological assessment for *Strongyloides* sp. is required to achieve satisfactory results, as opposed to a single stool examination.

## Introduction

Gastrointestinal parasite (GIP) infections, both helminthic and protozoan, are a major cause of global morbidity. It has been estimated that up to a quarter of the global population may be infected with intestinal helminths [1] and that 20% of migrants arriving in the UK from endemic countries may have a helminth infection [2]. Despite extensive efforts, which have had significant impact, soil transmitted helminths (STH) were still estimated to cause 2 million DALYs lost worldwide in 2019 [3]. Parasitic intestinal protozoa affect hundreds of millions of people globally each year–*Giardia duodenalis* alone is estimated to cause diarrhoea in 200–300 million people [4,5] and *Entamoeba histolytica* may be responsible for up to 100,000 deaths annually [6]. In endemic areas, GIP infections are an important cause of malnutrition, particularly affecting children and leading to stunted growth and cognitive impairment.

Some GIPs can persist for years or even decades–*Strongyloides stercoralis* infection can be lifelong due to autoinfection, potentially leading to a fatal hyperinfection syndrome in the immunosuppressed, which is unfortunately often diagnosed incidentally. The global prevalence of *S*. *stercoralis* has likely been underestimated and may exceed 300 million or even 600 million people [7,8]. *Entamoeba histolytica* infections can have an incubation period of months to years before presenting as dysentery or amoebic liver abscess.

There are limited data on migrants arriving to the UK from endemic areas, particularly in asymptomatic or otherwise healthy individuals, and there are few established screening programmes. Internationally, few recent large data sets on migrant screening exist. A meta-analysis of migrant screening including 88 studies showed a pooled strongyloidiasis seroprevalence of 12.2% and stool-based prevalence of 1.8%; schistosomiasis seroprevalence was 18.4% and stool-based prevalence was 0.9% [9].

In the UK a screening programme has been in place since 2012 for personnel recruited from Nepal for the Gurkha regiments of the United Kingdom Armed Forces. Historical data had suggested high rates of helminth infection in this population [10], supported by more recent work showing a GIP prevalence of 21.7% [11,12]. In this retrospective study, we review the data from 9 years (up to 2020) of screening 2,263 members of this large, homogenous, well controlled population of healthy and economically active migrants from a GIP endemic location, namely Nepal.

## Methods

This was a retrospective analysis of data from screening male adults (18–21 years) who had completed a physically arduous selection process in Nepal and recently arrived in the UK. All recruits arrived in the UK in Spring (February-March) to one location and had no reported symptoms of GIP infection. Samples were taken as part of a routine medical examination for military service, and verbal consent was obtained from each participant prior to sample collection. Participants received a medical review and treatment for pathogens detected by this screening programme.

Samples were analysed at the Clinical Diagnostic Parasitology Laboratory (CDPL) at the Liverpool School of Tropical Medicine (LSTM) and records of sample results were obtained from this laboratory.

### Stool analysis

Three separate faecal samples were obtained from every participant at weekly intervals. Faecal samples were typically processed within 18–24 hours of passage and were stored and transported at room temperature. Each sample was processed as follows:

1. Formalin-ethyl acetate (FEA) concentration and light microscopy: One to two grams of faeces was added to 10ml of 10% neutral buffered formalin in water, vortexed and left to stand for up to 3 minutes. Faecal samples were concentrated using modified Ritchie’s formol-ether method with Evergreen faecal parasite concentrators. Following concentration, two coverslips of sample were examined by light microscopy for the presence of ova, cysts or larvae. The slides were examined by two experienced microscopists independently at x100 magnification, with abnormalities examined at x400 magnification. The presence of *Entamoeba* cysts was recorded; however, it is not possible to make a distinction between pathogenic (*E*. *histolytica*) and non-pathogenic species (E.g., *Entamoeba dispar*, *moshkovskii or bangladeshi*) via microscopy. Slides were stained using the modified Ziehl Neilson technique to identify *Cryptosporidium* or other coccidia, and iodine was used as an aid to light microscopy according to microscopist judgement, but no other staining process was routinely undertaken.
2. Faecal charcoal culture: Each sample underwent charcoal culture [13] and was incubated at 26 degrees Celsius for 6 days. Following this, samples were examined using x30 magnification for larvae of *Strongyloides* and hookworm species. Larvae were differentiated morphologically between *Strongyloides* and hookworm based on buccal cavity size for L1 larvae (short for *Strongyloides* vs long for hookworm) and tail-end shape for L3 larvae (forked for *Strongyloides* vs pointed for hookworm). Whilst differentiation between hookworm species is possible on L3 infective larvae [14], this was not routinely done as clinical laboratory practice.

### *Strongyloides stercoralis* ELISA

A sample of clotted blood was collected from all participants. An aliquot of serum was collected from each sample. IgG antibodies to *S*. *stercoralis* were detected by ELISA using standard protocols. Prior to 2016, all serum samples were tested using an in-house antibody ELISA designed by LSTM which detected IgG antibodies to *Strongyloides*. Antigen for this ELISA was obtained from pooled L3 *S*. *stercoralis* larvae collected from patient samples at the CDPL at LSTM. Diluted samples were added to a previously coated antigen plate and left to incubate. Following appropriate incubation with secondary antibody (goat anti-human IgG alkaline phosphatase conjugate) and substrate (para-nitrophenylphosphate), positive samples displayed a yellow colour whilst negative samples remained colourless. An optical density was obtained using a plate reader at 490nm wavelength. From 2016 onwards, all serum samples were analysed using a commercially acquired *Strongyloides* IgG ELISA produced by DRG International. Diluted samples were added to provided microwells coated with *S*. *stercoralis* L3 larvae antigen. Following appropriate incubation with enzyme conjugate (IgG) and chromogen substrate to the conjugate (TMB), positive samples displayed a yellow colour whilst negative samples remained colourless. An optical density was determined using a plate reader at 450nm wavelength. For both ELISAs, the threshold of positivity was defined as an optical density of ≥0.200. High negative values were defined as an OD between 0.185 and 0.199. Weak positives were defined as an OD between 0.200 and 0.249. A strong positive was defined as an OD ≥1.000. Both assays were validated using known positives, known negatives, known cross-reactive samples and samples used in a national laboratory evaluation scheme.

The study focused on GIP infections that are generally agreed to have pathogenic potential, as summarised in Table 1. *Entamoeba histolytica* complex (*E*. *histolytica*, *E*. *dispar*, *E*. *moshkovskii* and *E*. *bangladeshi)* were included. The presence of GIP protozoa known to be non-pathogenic, or of disputed pathogenicity, was not routinely recorded until 2015. These are not included in the detailed analyses presented here, apart from overall summary data. *Strongyloides stercoralis* infection was either proven directly via charcoal culture and light microscopy, or considered as possible infection in cases of positive serological testing i.e., detection of IgG via ELISA. Peripheral blood eosinophil counts were not routinely measured as part of the screening programme. Changes in prevalence of individual pathogens over time are presented with 95% confidence intervals, calculated using the Wilson/Brown hybrid method [15]. Associations between changes in prevalence and time were assessed using logistic regression and log-binomial regression (for total GIP infections) using SPSS V28 and GraphPad Prism 10 software.

**Table 1:** Gastrointestinal parasite (GIP) infections identified in 2,263 Nepalese Gurkha recruits who had recently migrated to the UK between 2012 and 2020, showing number of infections, percentage prevalence, and percentage of total infections by organism. It was not possible to differentiate between members of the *E*. *histolytica* complex using light microscopy.

Organism	No. of infections	% Prevalence	% of total infections
*Giardia duodenalis*	231	10.2%	44.0%
*Strongyloides stercoralis*: All tests	102	4.5%	19.4%
IgG ELISA positive*	99	4.4%	18.9%
Culture positives	4	<1%	<1%
Hookworm species	86	3.8%	16.4%
*Entamoeba histolytica* complex	46	2.0%	8.8%
*Trichuris trichiura*	27	1.2%	5.1%
*Hymenolepis nana*	20	0.9%	3.8%
*Ascaris lumbricoides*	6	<1%	1.1%
*Trichostrongylus* sp.	5	<1%	<1%
*Enterobius vermicularis*	1	<1%	<1%
Unidentified trematode ovum**	1	<1%	<1%
Total	525		

*Two different *Strongyloides* IgG ELISAs were used over the study period. See Methods for more details.

**Whilst not definitively identified, the ovum was noted to be large, oval in shape, and operculated; therefore most likely fasciolid.

## Results

**Fig 1:**
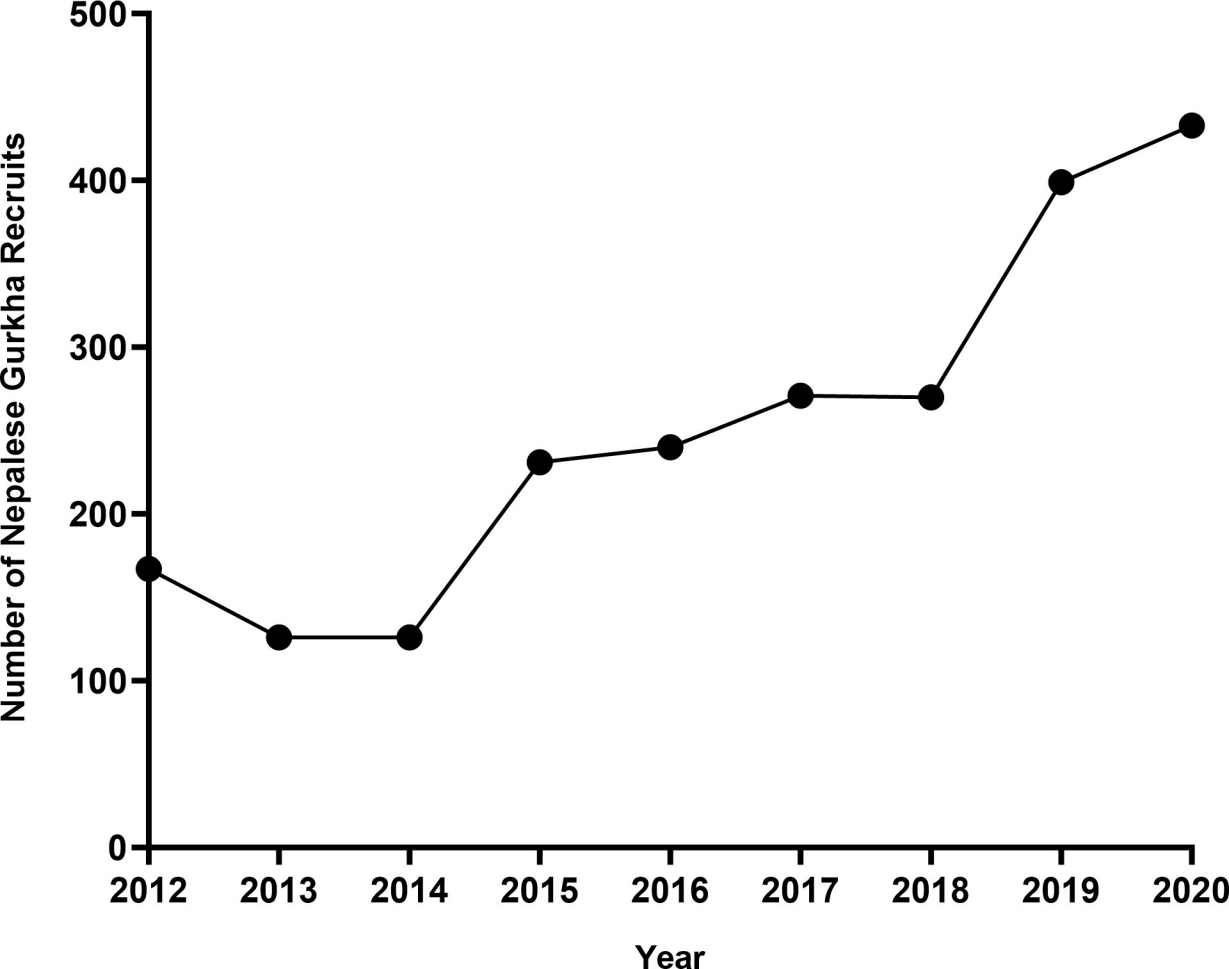
Number of Nepalese Gurkha recruits arriving in the UK and undergoing screening for GIP, each year from 2012–2020.

Results were available from 2,263 participants who completed screening between 2012 and 2020. The numbers of participants each year are summarised in Fig 1; the highest number screened was 433 in 2020.

### Infective organisms

In total, 463/2,263 (20.5%, 95% CI 18.8%-22.2%) participants had a positive diagnostic test for at least one infection with a known or potentially pathogenic GIP infection. A total of 525 such infections were identified in these 463 participants after combining all diagnostic methodologies (FEA concentration and microscopy, charcoal culture and serology), with a wide range of pathogens (Table 1). *Giardia duodenalis* was the most common organism identified with a prevalence of 10.2% (231/2263), making up 44.0% of identified infections (231/525). This was followed by *S*. *stercoralis*, with a positive diagnostic test in 4.5% (102/2263), making up 19.4% of infections (102/525). Third highest were hookworm species with a prevalence of 3.8% (86/2263), making up 16.4% of infections (86/525).

Of the 463 participants with an identified GIP infection, 409/463 (88.3%) were infected with a single organism, 46/463 (9.9%) were infected with two organisms, and 8/463 (1.7%) were infected with three organisms. The most common co-infections were with *G*. *duodenalis* and hookworm species (14 cases), *S*. *stercoralis* and hookworm species (13 cases) and *G*. *duodenalis* and *S*. *stercoralis* (12 cases). Three participants had a triple infection with *G*. *duodenalis*, *S*. *stercoralis* and hookworm species.

**Fig 2:**
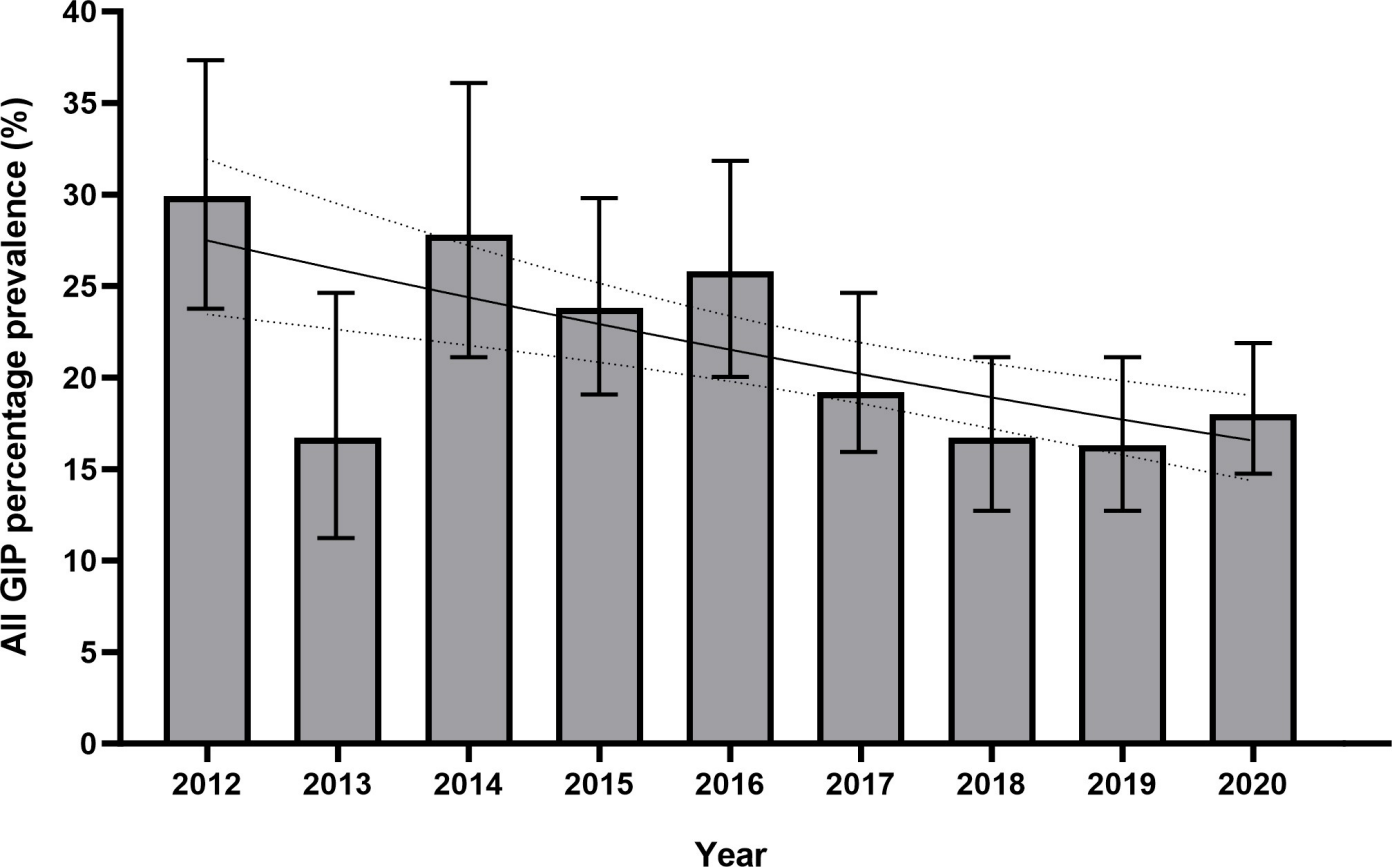
Bar chart and logistic regression curve of combined gastrointestinal parasite (GIP) infection prevalence in Nepalese Gurkha recruits who migrated to the UK by year, 2012–2020. Details of GIP infections identified are shown in Table 1. Logistic regression was statistically significant (p<0.001, OR 0.923, 95% CI 0.886–0.960) for a negative association with total GIP prevalence and time in years. If data for outlier year 2013 are excluded, this association is stronger (p<0.001, OR 0.903, 95% CI 0.865–0.942). Figures are %, bars show 95% CI. Logistic regression curve shown with 95% CI.

There was a negative association between overall GIP infection prevalence and time, falling from 29.9% in 2012 to 18% in 2020. A logistic regression found a reduction in the odds of a positive test for GIP falling by 7.7% (p<0.001, OR 0.923, 95% CI 0.886–0.960) for each year, and log-binomial regression found a reduction in the risk ratio of a positive test for GIP by 6.1% each year (p<0.001, RR 0.939, 95% CI 0.910–0.968). The year 2013 was identified as an outlier, with a lower-than-expected GIP infection prevalence of 16.5% (Fig 2).

### Protozoa

**Fig 3:**
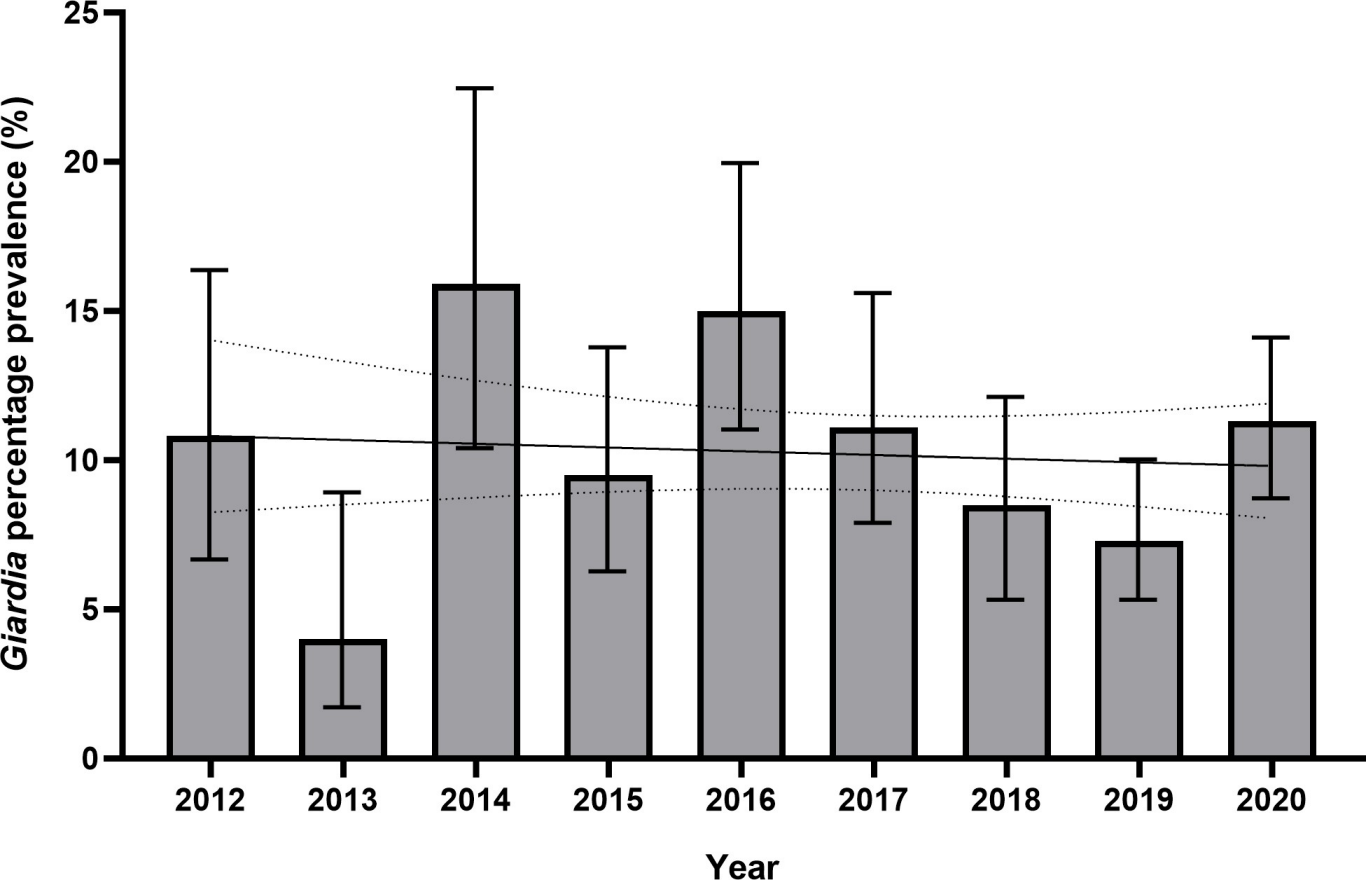
Bar chart and logistic regression curve of yearly prevalence of *G*. *duodenalis* infection prevalence in Nepalese Gurkha recruits who migrated to the UK 2012–2020. Logistic regression was not statistically significant (p = 0.625, OR 0.987, 95% CI 0.935–1.041) for an association between change in *Giardia* prevalence and time in years. Figures are %, bars show 95% CI. Logistic regression curve shown with 95% CI.

*Giardia duodenalis* was the most commonly identified pathogen, with 231 individuals found to have an infection (prevalence 10.2%, 95% CI 9%-11.5%) over the 9-year period, and accounting for 44% of infections identified. Unlike the trend seen in overall GIP infections, there was no evidence of a significant negative association in *Giardia* infection prevalence over time on logistic regression (p = 0.625, OR 0.987, 95% CI 0.935–1.041) (Fig 3).

It is not possible to differentiate between the pathogenic *E*. *histolytica* and non-pathogenic *E*. *dispar/moshkovskii/bangladeshi* on light microscopy. Overall, 46 individuals were found to have *E*. *histolytica or E*. *dispar/moshkovskii/bangladeshi* cysts (prevalence 2.0%, 95% CI 1.5%-2.7%). There was no evidence of a negative or positive association with time on logistic regression (p = 0.675, OR 1.026, 95% CI 0.911–1.154).

The detection of protozoa usually considered non-pathogenic was recorded since 2015. The overall prevalence of non-pathogenic protozoa was 29.7% (548/1844, 95% CI 27.6%-31.8%) from 2015–2020. Organisms identified include *Blastocystis* species (420/1844, 18.6%), *Endolimax nana* (250/1844, 11.0%), *Entamoeba hartmanni* (57/1844, 2.5%), *Entamoeba coli* (40/1844, 1.8%) and *Iodamoeba buetschlii* (30/1844, 1.3%) as well as a low prevalence of *Entamoeba polecki* (1/1844) and *Chilomastix mesnili* (3/1844). From 2015–2020, 41.4% (764/1844, 95% CI 39.2%-43.7%) of participants studied had either a pathogenic or non-pathogenic organism detected. There was a statistically significant positive association between detection of non-pathogenic protozoa and a positive test for a pathogenic organism, both for positive faecal methodologies only (OR 2.03, 95% CI 1.57–2.63), and by any positive test (i.e., inclusive of *S*. *stercoralis* IgG ELISA positivity) (OR 1.73, 95% CI 1.36–2.20). There was a negative association between non-pathogenic protozoa prevalence and time in years on logistic regression (p = 0.007, OR 0.923, 95% CI 0.871–0.979), and prevalence fell from 35.9% in 2015 to 26.8% in 2020.

### Helminths

**Fig 4:**
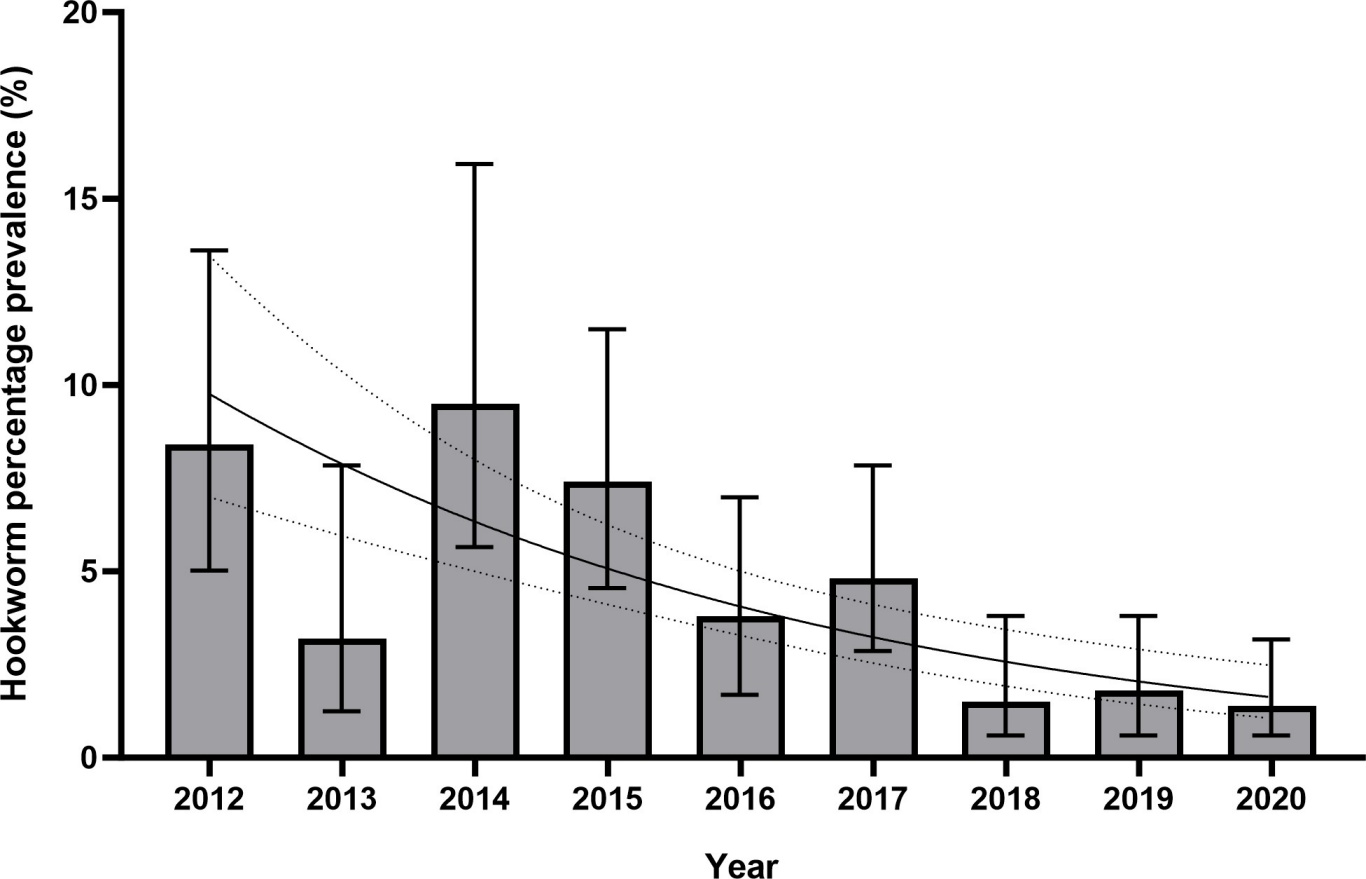
Bar chart and logistic regression curve of yearly prevalence of hookworm infection in Nepalese Gurkha recruits who migrated to the UK 2012–2020. Logistic regression was statistically significant (p<0.001, OR 0.791, 95% CI 0.729–0.860) for a negative association with hookworm prevalence and time in years. Figures are %, bars show 95% CI. Logistic regression curve shown with 95% CI.

Overall, 86 cases of hookworm infection were detected (prevalence 3.8%, 95% CI 3.1%-4.7%). Differentiation between hookworm species was not routinely done as laboratory practice. Hookworm infection prevalence fell from 8.4% in 2012 to 1.4% in 2020 and was not above 2% in any year since 2018. There was a negative association between hookworm prevalence and time; logistic regression found a reduction in the odds of a positive test for hookworm falling by 20.9% (p<0.001, OR 0.791, 95% CI 0.729–0.860) for each year of the study period (Fig 4).

**Fig 5:**
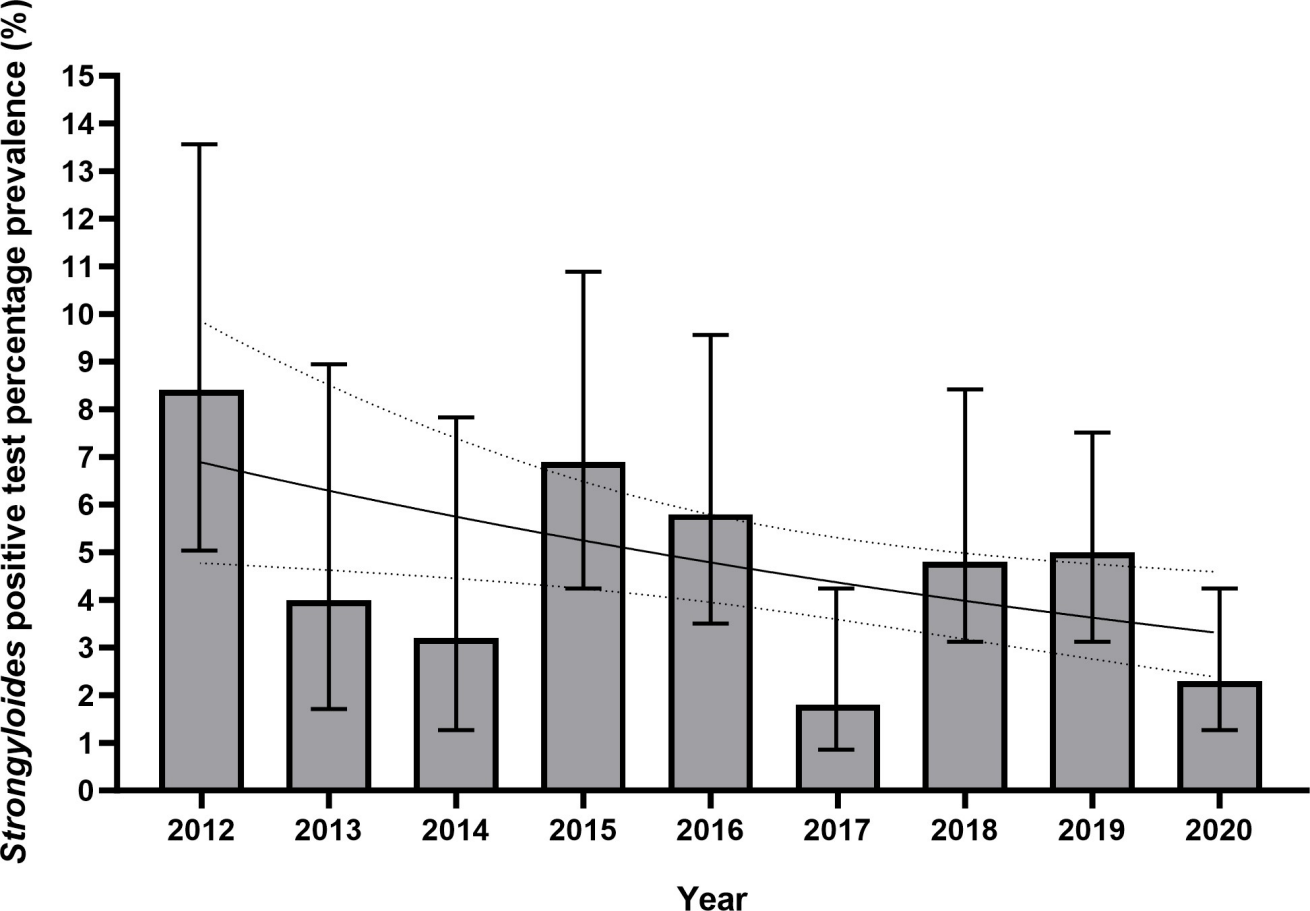
Bar chart and logistic regression curve of yearly positive *S*. *stercoralis* results (both charcoal culture and IgG ELISA), in Nepalese Gurkha recruits who migrated to the UK 2012–2020. Logistic regression was statistically significant (p = 0.013, OR 0.908, 95% CI 0.842–0.980) for a negative association with a positive *S*. *stercoralis* test and time in years. Figures are %, bars show 95% CI. Logistic regression curve shown with 95% CI.

Overall, 102 cases of proven/possible *S*. *stercoralis* infection were identified using serological methods or culture/microscopy (prevalence 4.5%, 95% CI 3.7–5.4). Ninety-nine participants had a positive *S*. *stercoralis* IgG ELISA result. Four participants were proven to have infection via identification of larvae in charcoal culture. In only one case was a positive ELISA result concordant with a culture result, in 2019. In the other three instances of positive charcoal culture, *S*. *stercoralis* IgG ELISA was negative. Of those that had a positive serological result, 10 were classed as a weakly positive result (Defined as an OD of 0.200–0.249), and 89 were classed as a positive result (OD 0.250–0.999). No *S*. *stercoralis* larvae were identified by FEA concentration and microscopy over the 9-year study period. There was a negative association between *S*. *stercoralis* positivity and time on logistic regression (p = 0.013 OR 0.908, 95% CI 0.842–0.980) (Fig 5).

**Fig 6:**
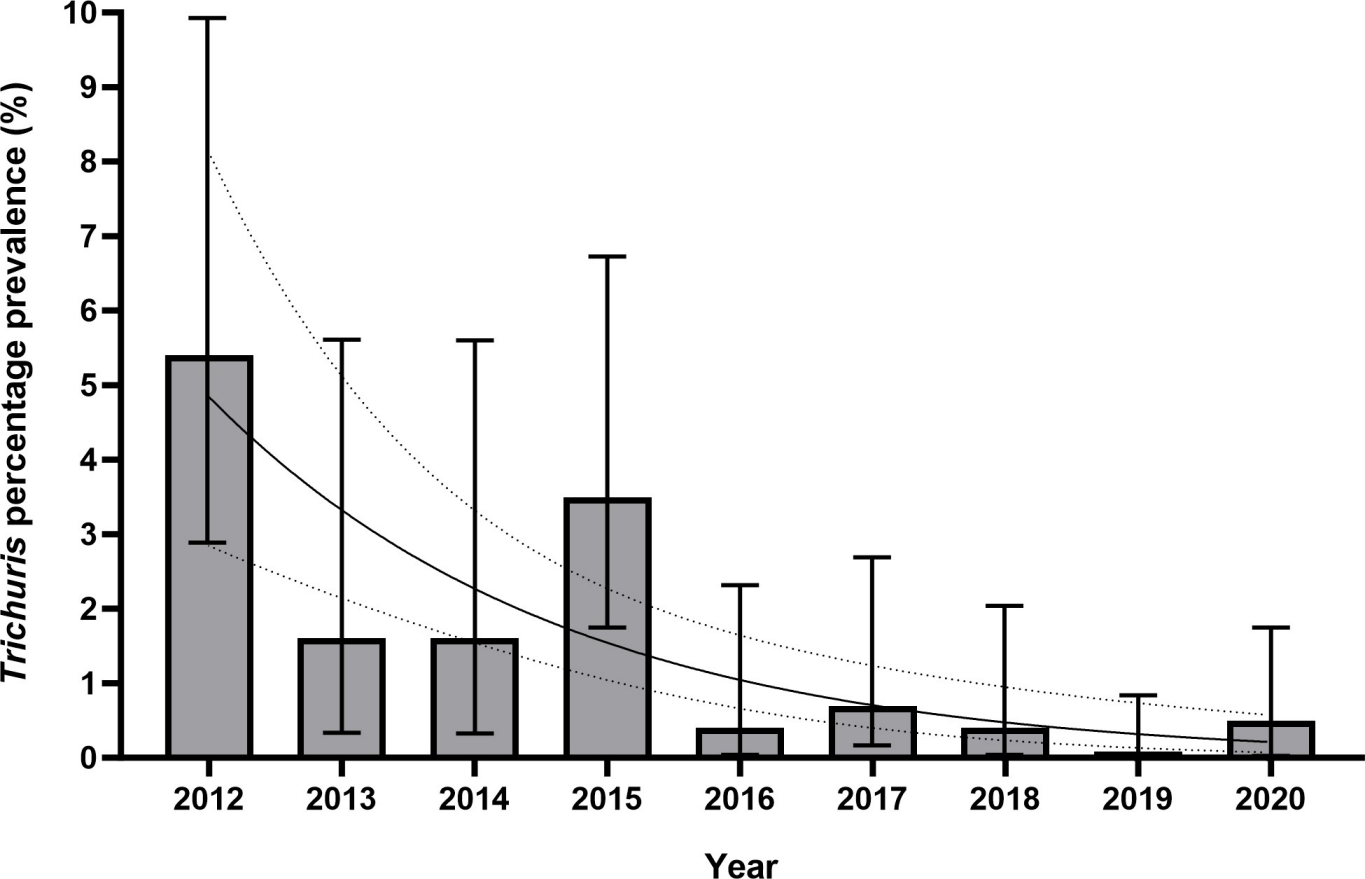
Bar chart and logistic regression curve of yearly prevalence of *T*. *trichiura* ova detection in Nepalese Gurkha recruits who have migrated to the UK 2012–2021. Logistic regression was statistically significant (p<0.001, OR 0.677, 95% CI 0.577–0.795) for a negative association with *T*. *trichiura* prevalence and time. Figures are %, bars show 95% CI. Logistic regression curve shown with 95% CI.

Far fewer cases of *Trichuris* infection were identified. All 27 cases were diagnosed via ova identification using light microscopy, with an overall prevalence of 1.2% (95% CI 0.8%-1.7%). Prevalence was much higher in the first year studied at 5.4% (9/167, 95% CI 2.9%-9.9%), and generally fell in subsequent years apart from 2015 when it was 3.5% (Fig 6).

### Other helminths

Twenty cases of *Hymenolepis nana* (dwarf tapeworm) infection were found, giving an overall prevalence of 0.9% (95% CI 0.6%-1.4%). Unlike *T*. *trichiura* and hookworm, there was no significant negative association of *H*. *nana* infection with time in years (Fisher’s exact test p = 0.262). A small number of cases of *Ascaris lumbricoides* (6 total) and *Trichostrongylus* sp. (5 total) were found.

### Diagnostics

#### Formalin-ethyl acetate concentration and light microscopy

There were 339/2263 (15.0%) individuals who had at least one stool sample positive for a pathogen by FEA concentration and microscopy. Of those, 185/339 (54.6%) were pathogen positive in one stool sample only, 62/339 (18.3%) were positive in two samples, and 92/339 (27.1%) were positive in all three samples. In 210/339 (61.9%) a pathogen was identified on the first stool sample analysed. In 83/339 (24.5%) a pathogen was first detected on the second sample. In 46/339 (13.6%) a pathogen was identified on the third sample only. The cumulative percentage of positive diagnoses for the main pathogens identified by FEA concentration and microscopy dependent on whether one, two or three stool samples were analysed, is shown in Table 2.

**Table 2:** Cumulative percentage of positive diagnoses made by light microscopy following FEA concentration, depending on whether one, two or three stool samples were analysed, in Nepalese Gurkha recruits who migrated to the UK between 2012 and 2020: i. By % and number of participants with at least one positive result on FEA concentration and light microscopy. ii. By % and number of pathogen identifications. Some participants were infected by more than one pathogen, and thus numbers of participants and pathogens identified are not equal. Hookworm identifications are not included but can be found in Table 3. Non-pathogenic protozoa are not included in this table.

	Cumulative % positivity after number of stool samples		
	1 sample	2 samples	3 samples
i. Participants with at least one positive test on FEA concentration and light microscopy	61.9% (210/339)	86.4% (293/339)	100% (339/339)
ii. Identifications of:			
*Giardia duodenalis*	61.5% (142/231)	86.1% (199/231)	100% (231/231)
*Entamoeba histolytica* complex	50.0% (23/46)	76.1% (35/46)	100% (46/46)
*Trichuris trichiura*	55.6% (15/27)	85.2% (23/27)	100% (27/27)
*Hymenolepis nana*	75.0% (15/20)	90.0% (18/20)	100% (20/20)
*Ascaris lumbricoides*	50.0% (3/6)	66.7% (4/6)	100% (6/6)
*Trichostrongylus* sp.	60.0% (3/5)	80.0% (4/5)	100% (5/5)
*Enterobius vermicularis*	00.0% (0/1)	00.0% (0/1)	100% (1/1)
Unidentified trematode ova	00.0% (0/1)	100% (1/1)	100% (1/1)
Total	59.6% (201/337)	84.3% (284/337)	100% (337/337)

#### Charcoal culture

A total of 82/86 (95.3%) cases of hookworm infection were diagnosed by charcoal culture. Of those culture positive for hookworm, 49/82 (59.8%) were positive in one culture only, 25/82 (30.5%) were positive in two cultures, and 9/82 (11%) were positive in all three. Fifty percent (41/82) of charcoal culture diagnoses were made on the first stool culture, 26/82 (31.7%) were made on the second culture and 15/82 (18.3%) diagnoses were made on the third culture. The cumulative percentage of diagnoses of hookworm made, using either solely microscopy, charcoal culture, or both, for one, two or three stool samples, is shown in Table 3.

**Table 3:** Cumulative percentage of positive diagnoses of hookworm species, using either FEA concentration and light microscopy alone, charcoal culture alone, or both methodologies, depending on whether 1, 2 or 3 samples were analysed, in Nepalese Gurkha recruits who migrated to the UK between 2012 and 2020.

	Cumulative % positivity after number of stool samples		
	1 sample	2 samples	3 samples
FEA and microscopy	18.6% (16/86)	32.6% (28/86)	37.2% (32/86)
Charcoal culture	47.7% (41/86)	77.8% (67/86)	95.3% (82/86)
Microscopy plus culture	47.7% (41/86)	82.6% (71/86)	100% (86/86)

A total of 4 cases of *Strongyloides* were diagnosed by charcoal culture. In 2/4 cases (50%), *Strongyloides* larvae were found in one culture only, and in the other 2/4 larvae were found in two cultures. In all 4 cases, there was co-infection with hookworm. In 3/4 cases (75%), *Strongyloides* larvae were found in the first culture sampled, and in 1/4 case, larvae were not found until the third culture.

## Discussion

The overall level of potentially pathogenic GIP infection in this population was high, at 20.5%. There has been a downward trend in infections since screening began in 2012, driven predominantly by a fall in helminth infections, most notably hookworm species. In contrast, infections with the protozoa *G*. *duodenalis* and *E*. *histolytica* complex have remained stable over time.

Previous studies conducted in Nepal have reported a range of prevalences of soil transmitted helminths; between 3.5 and 51.5% in adults [16], and 20.5% in school age children in the community [17]. High rates of protozoal infection have also been described; in a recent cross-sectional study of Nepalese schoolchildren aged 11–15, the prevalence of *G*. *duodenalis* was 21.1% and *E*. *histolytica* complex 12.6% [18]. In another study, *G*. *duodenalis* prevalence was 13.4% in children aged 6–14 [19]. Infection rates are not always as high as those found in the current study: a recent study of 400 prisoners in Kathmandu showed an overall parasite prevalence of 6%, with *Giardia* (2.5%) and *Trichuris* (1.5%) the most prevalent protozoa and helminth respectively [20].

Reasons for the fall in prevalence of helminth infection over time are unclear, but similar falls have been demonstrated in other geographical locations. In sub-Saharan Africa, the prevalence of STH in children is estimated to have fallen from 44% in 2000 to 18% in 2018, driven by a combination of anthelmintic medications, improved sanitation and economic development [21]. We have considered whether similar drivers exist in our population. Firstly, there is the possibility of overall increased hygiene and reduced exposure. The lack of soap for hand hygiene, lack of hand washing, and non-availability of toileting facilities have been found to be associated with a higher prevalence of both helminths and protozoa in previous studies in Nepal [16,18,22]. However, if improvements in hygiene and living standards were solely responsible for the drop in prevalence, it may be expected that there would be falls in all pathogens studied, but this was not observed for *G*. *duodenalis* or *H*. *nana*.

We also found no fall in the prevalence *of E*. *histolytica* complex. The majority of these are likely to be non-pathogenic *E*. *dispar/moshkovskii/bangladeshi*, or mixed infections, rather than pathogenic *E*. *histolytica*. Use of molecular identification techniques has revealed diversity in *Entamoeba* species in Nepal, with *E*. *histolytica* in the minority [23].

We did find a reduction in non-pathogenic protozoa detected, from 35.9% in 2015 to 26.8% in 2020. Whilst these are not widely accepted to be pathogenic to humans, their presence does indicate faeco-oral contamination. We found a positive association between the detection of non-pathogenic protozoa and detection of pathogens (OR 2.03, 95% CI 1.57–2.63). The detection of these commensal organisms may therefore have utility in identifying patients who should be investigated more thoroughly for possible pathogens.

A second potential explanation might be a change in patterns of recruitment of Nepalese Gurkhas, for example recruiting from a more affluent population, and/or from less rural areas, the implication being that those individuals may have access to footwear which would prevent percutaneous infection of hookworm or have occupations less likely to expose them to contaminated soil. Previous studies in Nepal have shown that a lack of wearing footwear outside was associated with increased prevalence [22] and intensity of hookworm infection [16], and that families who were engaged in farming were more likely to have GIP infections [18]. Whilst a change in recruiting pattern has been noted anecdotally, there are no published data to support this.

A third potential reason is the possible impact of Preventative Chemotherapy (PC) programmes that have taken place in Nepal. A programme to deliver albendazole to children aged 6–59 months twice a year was introduced in 1999, eventually reaching nationwide coverage by 2004 [24,25]. Albendazole has a higher cure rate for hookworm compared with *S*. *stercoralis* [26,27], potentially offering an explanation as to why hookworm prevalence has fallen at a higher rate than that seen for *S*. *stercoralis*. It is also highly effective against *A*. *lumbricoides*, which may explain the low prevalence of that helminth in this cohort. However, albendazole has low efficacy against *T*. *trichiura* [27], the prevalence of which fell during the study period (although the total numbers were low). PC may therefore not fully account for the fall in helminth prevalence. The prevalence of *G*. *duodenalis* did not fall with time, despite its susceptibility to albendazole, although treatment courses for *Giardia* are typically 3–5 days, rather than the single dose used for PC [28].

A further consideration is the possibility that due to increasing workload pressures and a decline in the number of experienced microscopists, a fall in recorded GIP infections may be indicative of a decline in detection rather than true prevalence. However, if that were the case, a fall in all pathogens detected via microscopy might be expected, whilst *Strongyloides* seropositive prevalence would remain static. However, as discussed, the prevalence of *G*. *duodenalis*, *H*. *nana* and *E*. *histolytica* complex has remained steady, while there has been a negative trend in *Strongyloides*. Furthermore, samples continue to be processed using the same methodology in a specialist laboratory which is subject to national external quality assurance.

Notably, we did not identify any cases of *Taenia* or *Cyclospora* infection, although both have been reported in Nepal [29,30]. The reasons for this absence are unclear, however, it has been suggested that *Taenia* infection may be more common in certain ethnic groups [29]. The highest incidence of *Cyclospora* infections has been reported as occurring in the Nepalese Summer (rainy season). Our study population arrived in the UK in early Spring, when infection rates are lowest [30].

Results in 2013 appear to be an outlier compared to the other years, with a GIP prevalence lower than expected. Removal of this year from the dataset provided a stronger negative association of GIP prevalence against time. We were not able to identify a clear reason for the lower-than-expected results in this year, although it is possible that participants may have been given treatment prior to their recruitment.

To our knowledge, this is the longest-running screening programme and largest published data set on pathogenic protozoa and helminths in Nepalese migrants anywhere in the world, and one of the few formal screening programmes for GIP infections in migrants to the UK from any endemic country. The generalisability of the results is somewhat limited by the study population, i.e., healthy young male participants. Other literature on parasitic infections in Nepalese migrants is extremely limited, and a recent meta-analysis of *Strongyloides* infection in migrants noted the paucity of studies in people from South Asia [31]. A study of migrant workers to Malaysia, which included 81 individuals from Nepal, found *G*. *duodenalis* was the most prevalent protozoan parasite, with a prevalence of 17.3%, similar to our data. However, they reported a much higher prevalence of helminth infections compared to our findings, with prevalences of 16% for hookworms and 72.8% for *A*. *lumbricoides*. This may be due to differences between the two migrant populations; our population underwent an arduous physical selection process in order to enter military service, compared with migrants to Malaysia who were migrating for low and semi-skilled work in manufacturing, food services, agriculture and plantation, construction and domestic services [32].

With the exception of the early work by O’Shea et al. in this population [11,12], there are limited recent published data on GIP infection in otherwise healthy or asymptomatic migrants to the UK. A large study in 4,000 schoolchildren conducted in the 1970s found a helminth prevalence of 30–40% in those migrating from Pakistan or India, but no participants were recorded as coming from Nepal [33]. Most recent studies have been performed in healthcare or hospital settings and have screened only those found to have an eosinophilia, potentially leading to an underestimate in overall population prevalence [34,35]. A study of migrant patients with eosinophilia in a London hospital, found that out of 201, 96 (48%) were diagnosed with *S*. *stercoralis* (via serology or faecal methods) and 9 (4.5%) were diagnosed with other intestinal helminths [36]. The authors are aware of an existing programme, Respond, designed to meet the complex healthcare needs of Asylum Seekers in London which includes screening for a range of GIP [37].

Parasite infection intensity was not formally quantified, but in most cases was noted to be light. This study confirms the importance of analysing multiple samples when utilising widely available traditional diagnostic methods to identify GIP infection. Using FEA concentration, 38.1% of infected participants were only identified as having a pathogen/potential pathogen after examination of a second or third sample. The majority of individuals (54.6%) were positive on only one stool sample out of the three. The low sensitivity of microscopy, even when using a concentration technique such as FEA, is supported by a previous meta-analysis–sensitivities using FEA in low intensity settings for *T*. *trichiura*, hookworm and *A*. *lumbricoides* were 21.5%, 38.9%, and 51.3% respectively [38].

Charcoal culture for hookworm was diagnostic in around 50% on either the second or third stool samples, and the majority (58.9%) were positive for hookworm in only one sample. We found FEA concentration and light microscopy to be less sensitive than charcoal culture for diagnosis of hookworm; 54/86 (62.8%) cases of hookworm infection were detected only by charcoal culture; 28/86 (32.5%) were detected by both charcoal culture and FEA light microscopy. In only 4/86 (4.7%) cases was FEA light microscopy positive with a negative charcoal culture. FEA light microscopy only detected 18.6% of 86 hookworm infections in a single stool sample, rising to 37.5% after examining three stools.

We found an overall *S*. *stercoralis* prevalence of 4.5% using both serological and faecal methods. The meta-analysis by Asundia et al. (2019), described a pooled prevalence of *Strongyloides* seropositivity of 12% and 1.8% stool positivity in migrants from all locations. For migrants from South Asia, the prevalences of seropositivity and stool positivity were 4.9% and 0.2% respectively [28], similar to our findings. The majority of diagnoses were made by serological means alone, which may represent past exposure rather than current infection, or antibody cross reaction which has been recognised in those coming from countries which are endemic for other helminthiases [39]. This has been most notable in cases of lymphatic filariasis infection [40]. However, stool microscopy has a low sensitivity, even after FEA concentration. Culture methods, such as charcoal culture used in this study improve sensitivity, but potentially not enough to detect low-level chronic infections [41,42]. Without the use of serology, it is likely that even a multiple sampling approach such as used in this study would underestimate prevalence. Differentiating between false positives and true *S*. *stercoralis* infection may have been aided by the presence of an elevated eosinophil count. However, it is a limitation of our study that this test was not performed as part of the screening process.

To improve the screening pathway for Nepalese Gurkha recruits, a helminth/protozoa multiplex polymerase chain reaction (PCR) platform has been added to the diagnostic tests offered since 2021, with the aspiration to reduce the number of stool samples required without impairing diagnostic sensitivity. This will also have the advantage of being able to differentiate between *E*. *histolytica* and non-pathogenic members of the *E*. *histolytica* complex. Molecular techniques have increasingly been shown to have high sensitivity, as well as the advantage of rapid testing compared with culture [43]. Analysis of the data generated from this approach will provide large-scale information on the utility of molecular methods in migrant screening programmes.

## Conclusion

We presented the results of a 9-year GIP screening programme in healthy Nepalese Gurkha males migrating to the United Kingdom from 2012–2020. We found that while overall prevalence rates fell over time, rates of GIP infection remained high at the end of the study period, at 18%. The prevalences of hookworm, *S*. *stercoralis*, and *T*. *trichiura* all fell with time. However, the prevalence of *G*. *duodenalis*, *H*. *nana* and *E*. *histolytica* complex did not fall over time. The reason for the fall in helminth prevalence is unclear, but factors may include the impact of PC programmes and recruitment from more affluent groups. We also found a reduction in prevalence of non-pathogenic protozoa and time. Those with a non-pathogenic protozoa detected had twice the odds of having a positive test for a pathogen than those who did not (OR 2.03, 95% CI 1.57–2.63). We found only 61.9% of participants with a positive test on microscopy had a pathogen found in the first faecal sample studied. Our results demonstrate the importance of specialist parasitological techniques; 62.8% of hookworm positive participants were diagnosed using charcoal culture alone. In environments where traditional faecal study methodologies are the mainstay of diagnosis, we have demonstrated that multiple stool samples and specialist parasitological techniques are important to achieve acceptable sensitivity.
